# Correction: *NPR1*-like genes in *Theobroma cacao*: Evolutionary insights and potential in enhancing resistance to *Phytophthora megakarya*

**DOI:** 10.1371/journal.pone.0323957

**Published:** 2025-05-27

**Authors:** Muhammad Umar Rasheed, Aiman Malik, Muhammad Zeshan Haider, Adnan Sami, Muhammad Shafiq, Qurban Ali, Muhammad Arshad Javed, Ansar Ali

In the Conclusion section, there are references omitted from the second sentence of the first paragraph.

The correct sentence is: The NPR1 protein in *Arabidopsis* plays a pivotal role in activating the plant’s immune system, particularly in response to pathogen attacks. This activation involves the synthesis of SA and establishing SAR, which helps protect the plant from subsequent infections (Fister., et al 2018, Shi., et al 2013, Pokou., et al 2019).

The references are: Fister, A. S., Landherr, L., Maximova, S. N. & Guiltinan, M. J. Transient Introduction of CRISPR/Cas9 Machinery Targeting TcNPR3 Enhances Defense Response in Theobroma cacao. Frontiers in Plant Science. 2018;9. https://doi.org/10.3389/fpls.2018.00268.

Shi, Z., Zhang, Y., Maximova, S.N. et al. TcNPR3 from Theobroma cacao functions as a repressor of the pathogen defense response. BMC Plant Biol 13, 204 (2013). https://doi.org/10.1186/1471-2229-13-204.

Shi Z, Maximova S, Liu Y, Verica J, Guiltinan MJ. The salicylic acid receptor NPR3 is a negative regulator of the transcriptional defense response during early flower development in Arabidopsis. Mol Plant. 2013 May;6(3):802-16. doi: 10.1093/mp/sss091. 2012 Sep 17. PMID: 22986789.

Pokou DN, Fister AS, Winters N, Tahi M, Klotioloma C, Sebastian A, Marden JH, Maximova SN, Guiltinan MJ. Resistant and susceptible cacao genotypes exhibit defense gene polymorphism and unique early responses to Phytophthora megakarya inoculation. Plant Mol Biol. 2019 Mar;99(4-5):499-516. doi: 10.1007/s11103-019-00832-y. 2019 Feb 9. PMID: 30739243.

In the Discussion section, a reference is omitted from the first sentence of the third paragraph.

The correct sentence is: The evolutionary history of TcNPR genes can be deciphered through comparative genomic analysis across species (Argout., et al 2011).

The reference is: Argout, X., Salse, J., Aury, JM. et al. The genome of Theobroma cacao. Nat Genet 43, 101–108 (2011). https://doi.org/10.1038/ng.73.
