# Supplementary material for: NPR1-like genes in Theobroma cacao: Evolutionary insights and potential in enhancing resistance to Phytophthora megakarya
Source: PLoS One. 2025 Feb 14;20(2):e0318506. doi: 10.1371/journal.pone.0318506 (PMC11828391; doi:10.1371/journal.pone.0318506)
Supplement: Attachment — Submitted filename: Response to Reviewers.docx [file pone.0318506.s004.docx]

Response to Reviewers

5. Review Comments to the Author

Reviewer #1: 1. The manuscript needs major English language revision. A number of typo and grammatical mistakes have been seen in your manuscript, Please try to improve the readability of your manuscript as possible by consulting with an English language expert.

Response: Thank you to the reviewer for comprehensively reviewing our manuscript. The grammatical errors and language have been updated in the new version.

2. Abstract should be more precise. In Abstract, briefly explain how study was conducted and what measured.

Response: The abstract has been revised and updated.

3. Figure 1: The values of relationship and similarity index are missing. Try to improve the figure quality and please indicate the species names in the figure legend.

Response: These values of bootstrap and figure legend are updated.

4. One limitation of this study is the reliance on the previous published data for gene expression profiling of Cacao varieties responses to Phytophthora Megakarya inoculation and the absence of experimental validation.

Response: We sincerely thank the reviewer for their valuable comments; however, due to limited resources, our study could not do the experimental work.

Reviewer #2: GENERAL

The authors used in silico approach to identify NPR-like genes in Cocoa and analyzed the implications especially in relation to their possible role in enhancing resistance to Phytophthora megakarya. No laboratory experiment was carried out.

I found that given the nature of the study, the appropriate software was used for analysis, and the results presented were largely reflective of the software output. There was no "big" need for the traditional statistical analysis except in Fig. 8 where I have commented below that the authors should state exactly what analysis was done since they derived raw data from a previously published paper.

Other section-by-section comments are compiled below:

============

INTRODUCTION

1. Overall, the manuscript is REPLETE with poor grammatical constructions that have substantially impeded logical flow and understanding of the work presented. A few examples are found in lines 49, 53, 68-69, 88, 90-91, 107-110, etc. The paper MUST NOT be accepted in the present form without a careful and extensive English language check and thorough revision. Also, attention should be given to standard scientific formats such as italicizing scientific names, capital letters beginning genus names and small letters beginning species name, writing the full form of an abbreviation at first mention (lines 70, 88, etc).

Author Response: The new version has resolved and updated these mistakes.

2. There are too many old references in the introduction and some of them do not seem to connect with the statements preceding them. For example, in lines 59-61 and 78-80, the authors made mention of expressions like "recent studies" and "until now" but cited references as old as "2004" and "2009". It would suggest that adequate literature review was not done to build in new references and connect them smoothly with the older references. Currently, the older references in the manuscript seem to have been merely imported from a previous similar paper.

Author Response: The new version has been updated and has resolved these mistakes and references.

3. Lines 78-87: It is quite confusing whether the authors were reviewing a past literature or talking about the current study under consideration since they kept using "we/our". The entire construction needs review.

Author Response: These lines have been rewritten for clarity.

MATERIALS AND METHODS

1. Section title should be: "Materials and Methods", not "Material and Methods"

Author Response: This typo has been corrected.

2. Be consistent with T. cacao, not T. cocoa or T. Cacao

Author Response: We sincerely apologize for the confusion. These mistakes have been resolved and updated in the new version. T cacao is the scientific name, while T cocoa is the processed one.

3. TcNPR-like sequences and not TcNPR like sequences (line 123-124).

Author Response: These mistakes have been resolved.

4. BTB domain means what?...line 124

Author Response: BTB is explained in the updated version.

5. Line 130-131: Any special reason for the choice of species whose NPR amino acid sequences were used in the phylogenetic analysis with TcNPRs?

Author Response: There is no specific reason; the few common species were used to compare the phylogeny.

6. Line 135: Do you mean Physico-chemical?

Author Response: It is physiochemical.

7. Lines 139-140: ...locations of chromosomal what???

Author Response: These mistakes have been resolved, and the sentence has been rewritten for clarity.

8. Line 141: "...was used to assess..." does not sound right. Recast.

Author Response: This sentence is revised.

9. Line 143: CERs or CREs? At first mention, state full meaning.

Author Response: The new version has resolved and updated these mistakes.

10. Lines 150-152: Statement does not make a lot of sense. Review and re-present.

Author Response: It has been corrected.

11. Lines 163-164: I think the sentence can be re-presented like this: "To investigate the expression levels of T. cacao NPR genes in response to Phytophthora megakarya inoculation, 2 variants of cocoa plants (SCA6 and NA32) were utilized".

Author Response: The new version has resolved and updated these mistakes.


RESULTS

1. Lines 221-223: Poor construction. Recast.

Author Response: These sections have been revised.

2. Lines 223-224: what does "thrice" mean in the context of its use?

Author Response: We sincerely apologize for the confusion; it has been updated.

3. Lines 278-279: The statement: "In T. cacao no gene happened to be a singleton as no duplication was found via synteny analysis" is somewhat confusing. If there was no duplication, does it not suggest that the genes exist as singletons?? Please clarify!

Author Response: The statement has been updated and clarified.

4. Line 290: It is unclear what analysis the authors exactly did under expression profiling. From the presentation, the transcriptomics data was obtained from a previously published report. So, what exactly did the authors do with this data to generate Fig. 8?

Author Response: We tried explaining this section in our updated manuscript version.


DISCUSSION

1. There are too many poor constructions, bad punctuations, and illogical flow. Please review extensively.

Author Response: This section has been revised extensively.

FURTHER CONSIDERATIONS

1. The authors should relook at this section. It would appear they are merely stating what they should have done instead of leaving it up to the readers to supply those information. In fact, as it stands currently, it seems as though the points raised under this section were for the authors to fill in before sending out the manuscript but somehow forgot to attend to them. This section should be re-done.

Author Response: This section has been removed and revised to a new version.
