# Supplementary material for: NPR1-like genes in Theobroma cacao: Evolutionary insights and potential in enhancing resistance to Phytophthora megakarya
Source: PLoS One. 2025 Feb 14;20(2):e0318506. doi: 10.1371/journal.pone.0318506 (PMC11828391; doi:10.1371/journal.pone.0318506)
Supplement: Attachment — Submitted filename: Response_to_Reviewers_auresp_2.docx [file pone.0318506.s005.docx]

Response to Reviewers

Review Comments to the Author

Reviewer #1: I appreciate the efforts made by the authors to improve the readability of the manuscript in the revised version. Now the manuscript can be accepted for publication after a minor revision.

1. In abstract, state the full name of the gene NPR1 at first mention.

Authors Response: We would like to thank the reviewer for their time and comprehensively improving the quality of our manuscript. The full name of NPR1 is mentioned.

2. Lines 25-27, Statement does not make a lot of sense, review and re-present.

Authors Response: The sentence has been restructured.

3. Lines 52-53, Poor construction, rewrite the statement.

Author Response: This sentence has been rewritten.

4. Lines 64-65, Are pathogens susceptible or cocoa plants are susceptible to pathogens? Please correct the statement.

Authors Response: We apologize for the mistake, it has been corrected in the new version of manuscript.

5. Conclusion section should be as concise as possible and should not be more than one or two paragraphs.

Author’s Response: The conclusion section has been trimmed and rewritten to make it more concise.

Reviewer #2: There has been a major English language revision in the manuscript and it is currently much better to understand. Also, most of the issues raised in the first review seem to have been addressed.


However, a few observations are noted below;


1. Lines 41-44: Authors referred to "Recent investigations" yet cited references as old as 1999 and 2004. This should be addressed.

Author’s Response: We thank the reviewers for pointing out the mistake; the new manuscript contains the updated references.

2. Line 257: The last sentence: "However, only one duplication is found in..." is incomplete.
Author Response: This sentence was missed, and we apologize to the reviewer; it has been replaced.

3. Physiochemical or physicochemical? Be consistent. See line 294

Author Response: The word has been changed to make it more consistent.
